# Prediction of internalizing and externalizing symptoms in late childhood from attention-deficit/hyperactivity disorder symptoms in early childhood

**DOI:** 10.1017/S0954579424000695

**Published:** 2024-03-27

**Authors:** Agnieszka Mlodnicka, Maxwell Mansolf, Aruna Chandran, Izzuddin M. Aris, Catrina A. Calub, Shaikh Ahmad, Allison Shapiro, David Cochran, Bibiana Restrepo, Rebecca Schmidt, Irva Hertz-Picciotto, Deborah Bennett, Diane R. Gold, T. Michael O’Shea, Leslie Leve, Julie B. Schweitzer

**Affiliations:** 1 Department of Psychiatry and Behavioral Sciences, University of California, Sacramento, CA, USA; 2 MIND Institute, University of California Davis, Sacramento, CA, USA; 3 Department of Medical Social Sciences, Northwestern University Feinberg School of Medicine, Chicago, IL, USA; 4 Bloomberg School of Public Health, Johns Hopkins University, Baltimore, MD, USA; 5 Department of Population Medicine at the Harvard Pilgrim Health Care Institute and Harvard Medical School, Boston, MA, USA; 6 Division of Developmental Medicine University of California San Francisco, San Francisco, CA, USA; 7 Department of Pediatrics, University of Colorado Anschutz Medical Campus, Denver, CO, USA; 8 Department of Psychiatry, Eunice Kennedy Shriver Center, UMass Chan Medical School, Worcester, MA, USA; 9 Department of Pediatrics, University of California Davis, Sacramento, CA, USA; 10 Department of Public Health Sciences, University of California Davis, Davis, CA, USA; 11 Channing Division of Network Medicine, Brigham and Women’s Hospital, Harvard Medical School; Harvard T.H. Chan School of Public Health. Boston, MA, USA; 12 Department of Pediatrics, University of North Carolina at Chapel Hill, Chapel Hill, NC, USA; 13 Prevention Science Institute, University of Oregon, Eugene, OR, USA

**Keywords:** ADHD, conduct symptoms, depression, puberty, sex

## Abstract

Limited analyses based on national samples have assessed whether early attention-deficit/hyperactivity disorder (ADHD) symptoms predict later internalizing and externalizing symptoms in youth and the influence of sex and pubertal timing on subsequent psychiatric symptoms. This study analyzed data (*n* = 2818) from the Environmental influences on Child Health Outcomes Program national cohort. Analyses used data from early childhood (mean age = 5.3 years) utilizing parent-reported ADHD symptoms to predict rates of internalizing and externalizing symptoms from late childhood/adolescence (mean age = 11.9 years). Within a subsample age at peak height velocity (APHV) acted as a proxy to assess pubertal timing from early childhood (mean age = 5.4 years) to adolescence (mean age = 12.3 years). Early-childhood ADHD symptoms predicted later psychiatric symptoms, including anxiety, depression, aggressive behavior, conduct problems, oppositional defiant disorder, and rule-breaking behavior. Earlier APHV was associated with increased Conduct Disorder symptoms from late childhood to adolescence for females only. A stronger relation between ADHD symptoms and later aggression was observed in females with earlier APHV, whereas this same pattern with aggression, conduct problems and depression was observed in males with later APHV. Clinicians should consider that both young girls and boys with elevated ADHD symptoms, particularly with off-set pubertal timing, may be at risk for later psychiatric symptoms.

## Introduction

ADHD is a neurodevelopmental disorder consisting of a constellation of symptoms designated as either inattentive or hyperactive/impulsive, or a combination of symptoms from both domains (American Psychiatric Association, 2013). The estimated prevalence of ADHD in youth in the U.S. is 9.4% (Danielson et al., 2018), with boys being diagnosed twice as often as girls (Willcutt, 2012). For many children, ADHD symptoms persist into adulthood (Barkley, 2015; Sibley et al., 2021), with a global prevalence of 2.6% in adults (Song et al., 2021). ADHD in school-aged children is associated with greater discord with parents and peers and academic underachievement (Barkley, 2015). In adolescence and young adulthood, ADHD is associated with increased rates of accidents, substance use, suicidality, and higher mortality (Ruiz-Goikoetxea et al., 2018; Sun et al., 2019). In addition, ADHD has high comorbidity with other disorders, including oppositional defiant (23%), conduct (27%), anxiety (12%–25%), and depressive disorders (14%) (Larson et al., 2011; Reale et al., 2017; Reimherr et al., 2017). Longitudinal studies find that childhood ADHD diagnosis and elevated symptoms predict future externalizing symptoms, such as oppositional defiant, conduct, and substance use disorders (Barkley et al., 1990; Bell et al., 2022; Klein et al., 2012; Noren Selinus et al., 2016). A population-based study by Noren Selinus et al., (2016) discovered that girls with ADHD symptoms in early childhood and adolescence experienced higher rates of internalizing problems compared to boys. Furthermore, the severity of ADHD symptoms in early childhood predicted the severity of adolescent internalizing symptoms in girls, but not in boys.

### Sex differences, comorbid symptoms, and longitudinal trajectory in ADHD

The ADHD research landscape, historically skewed towards male-centric studies due to the disorder’s higher prevalence in boys (Willcutt, 2012), is now evolving. Recent research efforts have increasingly focused on ADHD in female cohorts. For instance, the Hinshaw laboratory (Gordon & Hinshaw, 2020; Hinshaw, 2018) has been instrumental in demonstrating that females with childhood ADHD diagnoses are more susceptible to a range of adverse outcomes, including suicide attempts, self-injury, academic difficulties, internalizing disorders, and executive dysfunction, when compared to their non-ADHD counterparts (Gordon & Hinshaw, 2020; Hinshaw et al., 2012; Hinshaw, 2018). Similarly, other studies show that girls who meet criteria for ADHD face greater psychiatric comorbidity, behavior problems, and functional impairments than girls in ADHD remission (Mick et al., 2011) and girls with subclinical or moderate levels of ADHD symptoms (Mowlem et al., 2019; Noren Selinus et al., 2016). These differences are not similarly observed in boys, perhaps due to differences in parental perceptions of ADHD symptoms and impairment in boys versus girls (Mowlem et al., 2019). A recent 8-year longitudinal study indicated that female youth had lower levels of hyperactivity, impulsivity, and inattention than males, but that females had higher levels of impairment than males (Eng et al., 2023). The study also found that depression symptoms were more common in youth with ADHD who were older or in advanced stages of puberty, especially in females. This suggests a potential link between ADHD, developmental progression, and mental health.

### Puberty, psychopathology, and ADHD

The interplay between puberty and ADHD also warrants attention. Puberty’s hormonal and brain changes (i.e., estrogen, testosterone, and dopaminergic reorganization) are theorized to affect neurodevelopment, leading to increases in risky behavior, impulsivity, and insufficient emotional control (Steinberg, 2008). This potentiates a unique window for vulnerability in development (Forbes & Dahl, 2012) and in symptoms associated with ADHD. Thus, we hypothesized that pubertal timing could moderate ADHD symptoms and their associated comorbid psychiatric symptoms. For example, higher levels of ADHD severity are associated with higher levels of depression in early maturing girls and later maturing boys (Babinski et al., 2019). Indeed, existing literature suggests a link between the timing of pubertal onset and psychopathology (Graber, 2013), including an association between early puberty onset in girls with greater depressive symptoms in early to mid-adolescence (Joinson et al., 2013). More specifically, behaviors and disorders frequently comorbid with ADHD, such as risky behaviors and sensation/novelty seeking, conduct disorder, oppositional defiant disorder, and aggression frequently emerge during adolescence, a developmental stage often co-occurring with puberty (Casey et al., 2008).

Studies exploring the relation between ADHD, pubertal development, and mental health present a complex picture. Greenfield, Hechtman, Stehli et al., (2014) did not find an association between ADHD diagnosis or stimulant medication use, which is commonly used to treat ADHD, and Tanner ratings of puberty although a trend was observed between stimulant medication use and later puberty from an auxological growth measure. Rosenthal and Hinshaw (2023) did not find significant pubertal timing differences overall comparing girls with and without ADHD, however they found evidence of an association between delayed menstruation in girls with ADHD using stimulant medication versus those with ADHD who did not use stimulant medication. The connection between the use of stimulant medication and the timing of puberty or the onset of menarche is currently uncertain. However, it is possible that this link exists because stimulant medication tends to decrease appetite (Waxmonsky, Pelham, Bawjea, et al., 2022), leading to a lower body mass index (Rosenthal & Hinshaw, 2023; Waxmonsky, Pelham, Bawjea, et al., 2022).

Pai et al. (2022) observed a higher incidence of precocious puberty in boys and girls with ADHD, girls overall, in youth living in more urbanized areas. However, no relation between taking ADHD medication and central precocious puberty (Pai et al., 2022) or sexual development within the ADHD cohort emerged. Currently, knowledge of the association between pubertal timing, early ADHD symptoms, and later comorbid symptoms is limited.

### Current study

Data from children participating in the National Institutes of Health (NIH) Environmental Influences on Child Health Outcomes (ECHO) Program provide the opportunity to explore the relation among ADHD symptoms, subsequent internalizing/externalizing symptoms, sex, and pubertal timing in a large, sociodemographically, and geographically diverse population. In this study, we analyzed ECHO cohort data to examine relations between ADHD symptoms in early childhood (i.e., 3–9 years of age) and internalizing and externalizing symptoms in middle childhood through adolescence (9–18 years of age). A better understanding of how pubertal timing affects the relation between ADHD symptoms in early childhood and later externalizing and internalizing symptoms will enable us to develop a more comprehensive understanding of ADHD symptomatology, comorbidities, prevention, and treatment plan development for youth with elevated ADHD symptoms. We used estimated age at peak height velocity (APHV), a surrogate and objective measure for pubertal timing (specifically, the age at which linear growth during puberty is fastest), to explore the relation between sex differences and pubertal timing, as sex differences in APHV are well established in the literature (Kelly et al., 2014; O’Neill et al., 2022; Zheng et al., 2013). We anticipate finding differences by sex in the impact of pubertal timing and how it may moderate the relation between ADHD symptoms and the later expression of internalizing and externalizing symptoms. Based on the findings of Babinski et al. (2019), we hypothesized that greater ADHD symptoms at an earlier age (3–8 years) would be associated with greater internalizing and externalizing symptoms in later childhood (9–18 years), regardless of sex. Furthermore, we hypothesized that earlier pubertal onset in both girls and boys would modify the effect of ADHD on internalizing and externalizing outcomes.

## Methods

### Participants

The ECHO Program is a nationwide consortium of child cohorts (Gillman & Blaisdell, 2018). Children who met the following criteria were included in the analysis: they were aged 3 to less than 9 years, completed a measure of internalizing and externalizing symptoms at the age of 9 years or older but not older than 18 years, had available data on sex, and were classified as either male or female. Cohorts lacking these measures were excluded from the analysis. A total of 21 cohorts contributed data to this analysis. The Institutional Review Boards of each cohort site, the Data Analysis Center (Johns Hopkins Bloomberg School of Public Health) host institutions, and the Patient-Reported Outcomes Core (Northwestern University) reviewed and approved the study activities.

### Measures

We selected measures of childhood ADHD symptoms and later internalizing and externalizing symptoms based on: (a) the availability of a parent-report measure of child symptoms; (b) the availability of standardized scores (*T*-score; mean = 50; standard deviation (SD) = 10 in the referenced sample) for the measure; and (c) the relevance of the measure, which must relate to ADHD symptoms and/or later outcomes (internalizing and externalizing disorders) domains. When calculating ADHD symptoms based on the content of the measure, we used a global ADHD symptom score composed of inattention and hyperactivity/impulsivity symptoms if the score could be generated by the measure. If not, we averaged available inattention and hyperactivity/impulsivity scores or used the domain score that was available if the other domain was missing for an individual. For internalizing and externalizing symptoms, we included subscales corresponding to the following domains: Aggressive Behavior (*E* = Externalizing), Rule-Breaking (E), Conduct Problems (E), Oppositional Defiant Problems (E), Depression (*I* = Internalizing), and Anxiety (I). We chose to include specific, narrower domains of children’s behavioral and emotional functioning over broadband domains for two reasons: 1) they provide more specificity for informing precision interventions; 2) requiring broadband measures would have restricted our sample size, as some cohorts only used narrower domain assessments (e.g., PROMIS depression or anxiety lacks a broadband scale). In accordance with these constraints, we describe the selected measures in the following sections (see Table 1 for a summary).

### Child behavior checklist

We used the caregiver form of the Child Behavior Checklist (CBCL) (Achenbach & Ruffle, 2000), including the version for ages 1.5–5 years (CBCL-Pre) and the version for ages 6–18 years (CBCL-Sch). The CBCL-Pre and CBCL-Sch are parent-reported questionnaires that contain 100 and 119 items, respectively. These items correspond to problematic behaviors observed within the past 6 months, with ratings from 0 (Not True) to 2 (Very True or Often True). We used both the syndrome and Diagnostic and Statistical Manual of Mental Disorders (DSM)-oriented CBCL scales to measure their corresponding domains (Table 1). Each syndrome and DSM-oriented scale yields a *T*-score, referenced to the U.S. general population. CBCL *T*-scores between 65 and 70 are considered borderline clinical, whereas *T*-scores greater than 70 are considered clinically significant.

### PROMIS v2.0 – depressive and anxiety symptoms

We used the PROMIS v2.0 - Depressive Symptoms 6a - Parent Proxy (6 items) and Anxiety 8a – Parent Proxy (8 items) short forms developed from their respective PROMIS Parent Proxy Item Banks v2.0. Both scales are parent reported. The PROMIS Depression items assess negative mood, views of self, social cognition, and decreased positive affect and engagement (Schalet et al., 2016). Somatic symptoms are excluded to avoid confounding effects in patients with comorbid physical conditions. The PROMIS Anxiety items assess fear, anxious misery, hyperarousal, and somatic symptoms related to arousal (Schalet et al., 2016). In both measures, items are rated on a scale from 1 (Never) to 5 (Almost Always) and use a 7-day reporting period. Both PROMIS short forms yield *T*-scores referenced to the U.S. general population.

### Behavior assessment system for children–parent rating scales

The Behavior Assessment System for Children, 2nd Edition (BASC-2) and 3rd Edition (BASC-3) are parent-reported, multidimensional forms capturing clinical and adaptive functioning (Reynolds & Kamphaus, 2015). The BASC is designed for three age ranges: preschool (2–5 years), child (6–11 years), and adolescence (12–21 years). The preschool version has 139 items, the child version 175 items, and the adolescent version 173 items. Items are rated on a 4-point scale, from 0 (Never) to 3 (Almost Always). Raw scores are summed and converted into standardized *T*-scores (mean = 50; SD = 10). *T*-scores between 60 and 69 are considered at-risk, and *T*-scores equal to or above 70 are considered clinically significant.

### Conners 3 ADHD index–parent

The Conners 3 ADHD Index–Parent (Conners 3AI–P) is a 110-item, parent-reported questionnaire designed to assess ADHD and associated symptoms (Conners, 2008, 2010). We used the 10-item ADHD Index screener within the larger scale, developed to distinguish children and adolescents (6–18 years of age) with ADHD from the general population. This scale, which includes representations of both Inattention (6 items) and Hyperactive/Impulsive symptoms (4 items), uses a 4-point Likert scale for each item, where higher scores indicate greater severity or frequency of concerns. Items on the 4-point scale range from 0 (Not at All) to 3 (Very Much True). The raw score is transformed into a standardized *T*-score. *T*-scores between 65 and 69 are considered at-risk, and *T*-scores equal to or above 70 are considered clinically significant.

### Domains and measure selection

Although not all cohorts used the same behavioral measures, these measures are well-matched, attempt to tap into the same processes, typically rely on DSM descriptors, and ask similar questions per domain. Using more than one measure for each construct suggests increased validity and generalizability in how these symptoms are measured across sites. We conducted separate analyses to evaluate the relationship between early ADHD symptoms and the seven domains, yielding slightly different sample sizes in each analysis. When multiple measures of ADHD, internalizing, or externalizing symptoms were available for an individual, we prioritized measures based on the relevance of each measure’s item content to our hypotheses, with the Conners 3AI–P questionnaire having the highest priority, followed by the CBCL, BASC, and PROMIS (in that order). All measures produce T-scores, which we treated as equivalent across measures in our analysis, ensuring every individual had a single ADHD symptom score and a single score for one or more of the seven outcome domains previously discussed. Where multiple scores were available for an individual and measure, scores were limited to one per individual by selecting the score obtained at an age closest to the median for each age range in the sample, breaking ties by selecting the form completed by the mother, father, or other caregiver (in that order).

### Age at peak height velocity

Linear growth typically accelerates during puberty due to the activation of the hypothalamic-pituitary axis. The timing of the pubertal growth spurt (i.e., APHV, the age at which linear growth during puberty is fastest) can serve as an objective marker of pubertal timing (Aris et al., 2019, 2022a; Carwile et al., 2021).

Furthermore, we obtained additional data on height from pediatric record well-child clinic visits. We previously estimated APHV in 34 ECHO cohorts (Aris et al., 2022) using longitudinal height data in children who had two or more height measurements from research visits and/or medical records (median [range] = 4 [2–19] height measurements per child; median [age range] = 8.4 [5–22] years). The mean (SD) APHV was 12.9 (0.4) years in males and 10.8 (0.5) years in females (Aris et al., 2022). Briefly, we estimated APHV by fitting subject-specific height growth curves using the SuperImposition by Translation and Rotation (SITAR) growth model, a non-linear mixed-effect model with estimation by maximum likelihood. SITAR uses a shape-invariant natural cubic spline curve and a non-linear random-effects model to estimate a population-average height growth curve for the entire sample and each participant’s deviation from the population-average curve as random effects. We identified the optimal model using the Bayesian information criterion. APHV for each child was estimated by differentiating the individually predicted height curves and locating the maximum inflection point during adolescence for each participant, where the derivative equals 0. In this study, we included APHV in all models as a continuous variable in units of years, centered separately by sex at the mean across all participants in our analytic sample for whom APHV in the ECHO Program was calculated (12.6 years for males, 10.6 years for females); note that this center was calculated in a different sample from the analyses presented (Aris et al., 2022). (See Table S1 for means and standard deviations of the time between initial ADHD symptomatology assessment and subsequent outcome assessment, separated by outcome domain and analysis sample.)

### Statistical analysis

Descriptive statistics for the included sample are presented in Table 2. As the specific analytic samples differed by outcome, the values in Table 2 include all individuals who were included in any analysis.

### General modeling strategy

Data were analyzed separately by outcome domain and sex, yielding separate sets of models for the Depression, Anxiety, Aggressive Behavior, Conduct Problems, Oppositional Defiant, and Rule-Breaking domains, with each set divided into models for males and females. For each outcome, the core statistical model predicted an outcome *T*-score from the ADHD *T*-score, with maternal education level included as a covariate with two levels: high school degree or below, and some college and above. We used multiple imputation to account for missing maternal education data. Data were imputed separately by cohort; thus, cohorts that did not have any data on maternal education were removed prior to analysis and were excluded from Table 2. For cohorts with fewer than 50 participants, we used listwise deletion to avoid unstable imputation models in these small samples. The mice package in R was used for imputation, with 100 imputations and 100 burn-in steps for each generated imputation.

We employed a random-effects model with a random intercept in which the ECHO cohort was used as the cluster variable. All models were estimated using restricted maximum likelihood (Raudenbush & Bryk, 2002) using the lme4 package (Bates et al., 2015). Each analytical model was run separately on each imputation, and the estimates were combined using Rubin’s pooling rules, yielding point estimates and confidence intervals (CIs) for each effect of interest (Rubin, 1987).

### Moderation analyses

For each outcome, we estimated two models. In the first model (Model A), the outcome *T*-score was predicted from the early-age ADHD *T*-score and maternal education (dummy-coded), with no other predictors included in the model (21 cohorts; N per analysis = [1288, 1441]). In the second model (Model B), APHV was included as an additional main predictor and as an interaction effect with ADHD. In Model B, only the subset of the sample with APHV estimates was included, yielding a smaller number of cohorts (*N* = 17) and a smaller total sample size in each analysis (*N* per analysis = [973, 1106]). The right-hand column of Table 2 contains descriptive statistics for the subsample included in any of the APHV moderation analyses. In each moderation analysis, the ADHD *T*-score was centered at 50 to facilitate interpretation of interactions; this *T*-score represents approximate population-average levels of ADHD symptoms.

## Results

In our primary analytic sample (*N* = 2,818), 51.4% (*N* = 1,448) of the participants were male, and 48.6% were female; 52.2% (*N* = 1,472) were White, and 31.9% (*N* = 898) were Black (Table 2). The majority (90.1%, *N* = 2,539) of caregivers reported non-Hispanic ethnicity. Nearly two-thirds (60.8%, *N* = 1,712) of the caregivers reported an annual household income of $50,000 or more. Most caregivers (75.2%, *N* = 2,119) reported having some college education or more. The demographic profile did not differ significantly among the subpopulation included in the APHV analysis (*N* = 2,160, Table 2). See the Supplemental Material for comparisons between our sample and the estimated prevalence in the U.S. according to Census data and references. The CBCL alone was used for the early measure and all outcomes in most of the sample (62.8% in the full sample, 57.6% in the APHV subsample). The BASC alone was used for another fraction (5.9% and 7.0%, respectively), and the remaining participants were measured using multiple instruments for the early measure and/or the multiple outcomes; see Supplemental Tables S2a and S2b for the frequencies of all combinations of measures used.

The results of the random-effects models are presented in Table 3. The coefficients for ADHD *T*-scores can be interpreted by Cohen’s *d* metric with respect to the general U.S. population; each 1-unit increase in ADHD *T*-score is associated with an estimated increase or decrease in each outcome *T*-score. Equivalently, each one-SD increase in ADHD *T*-score is associated with a proportion of an SD increase or decrease in each outcome *T*-score. This latter interpretation is possible because the SDs of the *T*-scores in the general population for the independent and dependent variables are the same (10 *T*-score points) by construction. In Model A, among both females and males, a one-SD increase in ADHD *T*-score is associated with a statistically significant 0.14–0.23 SD (1.4–2.3 *T*-score points) increase in internalizing and externalizing symptoms (i.e., anxiety, depression, aggressive behavior, oppositional defiant disorder, conduct disorder, rule-breaking). No significant differences (i.e., non-overlapping confidence intervals) were observed between females and males in any of the internalizing or externalizing symptoms.

When APHV was included as a predictor and moderator (Model B; see Table 4), APHV was found to be predictive of one externalizing domain—Conduct Problems (Est [CI] = −0.76 [−1.45, −0.07]), with each additional year of earlier APHV associated with a 0.076 SD (0.76 *T*-score points) increase in Conduct Problems in girls. In girls, estimates were lower for other externalizing symptoms but in the same direction as those for Conduct Problems (−0.19<Est < −0.67); however, these estimates were not statistically significant (*α* = .05, with the upper bound of CI between 0.02 and 0.42).

Significant measure modification was also found. Specifically, the estimated coefficient for the interaction of APHV and ADHD symptoms in females for Aggressive Behavior was −0.10 (CI = [−0.18, −0.01]), indicating that each one-year decrease in APHV (i.e., one-year earlier puberty) increased the association between ADHD symptoms and Aggressive Behavior by 0.10 *T*-score points. For males, the opposite was observed, wherein each one-year increase in APHV increased the association between ADHD symptoms and Aggressive Behavior by .06 (CI = [0.02, 0.11]) *T*-score points. A similar interaction was observed for Conduct Problems and Depression in males, wherein each one-year increase in APHV increased the association between ADHD symptoms and Conduct Problems by .05 (CI = [0.01, 0.09]) *T*-score points and between ADHD symptoms and Depression by 0.06 (CI = [0.01, 0.10]) *T*-score points. (See Supplemental Table 4 (S4) which contains, for each outcome and analysis sample, the proportions of males and females with outcome assessments after their estimated APHV. Due to earlier puberty (measured here by APHV), more females completed the outcome assessment after APHV than males.

## Discussion

### Summary of main findings

This study utilized data from the geographically and sociodemographically diverse ECHO Program to examine the relation between ADHD symptoms and later internalizing and externalizing behaviors, and whether sex and the age of maximum growth during puberty influence this relation. Our findings indicate that greater ADHD symptoms in early to middle childhood (3–9 years of age) were associated with increased internalizing behaviors (anxiety and depression) and externalizing behaviors (aggressive behavior, conduct problems, oppositional defiant disorder, and rule-breaking behavior) in later childhood and adolescence (9–18 years of age), regardless of sex. Moreover, some relations were more pronounced for females with early pubertal timing and males with later pubertal timing, suggesting these groups may be at higher risk for greater mental health problems.

### Later psychiatric outcomes

Our results corroborate earlier studies (Barkley, 2015; Biederman et al., 2006, 2010; Connor et al., 2003), which indicated that elevated ADHD symptoms increase the risk for later psychiatric symptoms. It may be possible that increased internalizing and externalizing behaviors are secondary to challenges associated with ADHD, including peer exclusion, poor self-esteem, poor academic performance, and family conflicts (Faraone et al., 2021; Johnston & Chronis-Tuscano, 2015). Given that the mean age for the early symptom measure (i.e., ADHD) for our participants was relatively young (5.3 years), our data suggest that even young children with elevated ADHD symptoms are at risk for additional psychiatric challenges, warranting consideration for the intervention of ADHD and any comorbid symptoms during preschool and kindergarten years.

The broader negative outcomes we identified could also be explained by the current conceptualization of ADHD as a disorder of regulation or ineffective cognitive and emotional control. Poor regulation could increase the likelihood of higher rates of comorbid psychiatric disorders and later internalizing and externalizing behaviors (Barkley, 2015). The results support further exploration of the theory of the “*p*” factor dimension, a general psychopathology dimension, where higher *p* scores are associated with higher psychopathology severity, uniting all psychopathology under one construct (Caspi & Moffitt, 2018). Thus, it may be that early ADHD symptoms are a manifestation of other psychiatric symptoms that have yet to emerge and are more reflective of a broad psychiatric vulnerability that becomes more evident in adolescence. However, because this analysis did not assess for the presence of internalizing or externalizing symptoms at the early time point, it may be that those symptoms were present early on and persisted or that they surfaced with later development. Future studies should include multiple, repeated measures from early childhood onward to determine whether the cascade or *p* factor theories are correct.

On a neural level, ADHD studies on brain structure and function may provide clues as to why we found higher ratings of behavioral dysfunction associated with early elevated ratings of ADHD symptoms. A growing body of evidence suggests that ADHD is associated with brain alterations that may have broader implications beyond ADHD symptoms. For instance, the disorder is linked with delayed maturation in the cortex (Shaw et al., 2014), particularly in the prefrontal cortex, smaller cortical surface areas (i.e., frontal, temporal, and cingulate regions) in younger children with ADHD, and smaller subcortical volumes (i.e., accumbens, amygdala, caudate, hippocampal, putamen, and intracranial) (Hoogman et al., 2019). Furthermore, ADHD is associated with differences in brain activity and connectivity between regions thought to control attention, impulsivity, and emotion, which could explain how dysregulation manifested as ADHD symptoms earlier in development could predict broader psychopathology at a later developmental period (Hoogman et al., 2017).

### Sex differences

Our findings indicate similarities in later externalizing and internalizing behavioral outcomes in girls and boys with early elevations in ADHD symptoms. These findings align with previous longitudinal studies (e.g., Bell et al., 2022) including a large study in Sweden (Noren Selinus et al., 2016) involving 4,635 participants, demonstrating an association between childhood ADHD symptoms and a range of adverse psychosocial outcomes in adolescence for both boys and girls. However, our findings diverge in one key aspect: the Swedish study observed elevations in internalizing symptoms only in girls with ADHD at a later time point, while we noted such increases in both sexes. Our results underscore the need for prevention, monitoring, and intervention for both externalizing *and* internalizing symptoms in both boys *and* girls, which may be under-recognized by parents and clinicians. Future research should investigate if prevention and treatment methods for externalizing symptoms in ADHD differ between girls and boys, as this could inform sex or gender-associated interventions for better outcomes.

### Pubertal timing

Using APHV as a predictor and effect modifier revealed a main effect on one significant domain (Conduct Problems) in girls only. With each additional year of earlier pubertal timing (measured via APHV), girls were more likely to be rated as displaying a significant increase in symptoms of Conduct Problems. We also found significant effect modifications for APHV on ADHD symptoms, such that there was an increased association between ADHD symptoms and aggressive behavior for earlier pubertal timing for females and later pubertal timing for males. Later pubertal timing in males was also associated with a stronger relation between ADHD symptoms and depression and conduct problems. Our finding that an earlier onset of age of APHV in girls was associated with higher rates of Conduct Problems is consistent with other findings that demonstrated earlier pubertal maturation in girls is associated with several detrimental outcomes, including elevated internalizing and externalizing symptoms and disorders and psychosocial difficulties (Babinski et al., 2019; Graber, 2013; Mendle et al., 2007; Ostojic & Miller, 2016; Wasserman et al., 2012. Earlier pubertal timing could influence the emergence of adverse psychosocial outcomes through several potential mechanisms, as it may precipitate a flurry of social changes for which girls may not be “developmentally ready.” As maturation inadvertently truncates the resolution of developmental tasks from the preadolescent period, girls may be forced to grapple with these social changes with fewer resources than later maturing peers. This includes other children and adults perceiving and acting toward them differently, expecting them to be more emotionally and psychologically mature based on their physical appearance. It is also possible that earlier pubertal timing may interact with hormonal changes in a way that may be particularly detrimental for girls who mature earlier. Increases in hormones, particularly estradiol, during puberty may heighten a girl’s sensitivity to social and academic conditions, resulting in disproportionate increases in negative mood and emotional states (Spear, 2009). This reasoning may lend insight into our findings that females with earlier pubertal timing who present with ADHD symptoms during middle childhood are at greater risk for later aggression. In contrast, our finding that males with later pubertal timing have a stronger relation between ADHD symptoms and other mental health problems (i.e., aggressive behavior, depression, conduct problems) is consistent with other studies that show late maturing boys experience higher rates of depressive symptoms and externalizing behaviors during adolescence (Babinski et al., 2019; Graber, 2013; Negriff & Susman, 2011).

### Strengths, limitations, and future directions

Our study had several strengths, including the use of objective, repeated measures of height and statistical models to estimate pubertal timing (Granados et al., 2015), and the inclusion of a large, relatively diverse national sample with similarly sized samples of boys and girls. Nonetheless, a number of limitations warrant consideration. Although clinical assessment of pubertal development (i.e., Tanner staging) by trained observers is considered the gold standard, it may also be prone to measurement error and bias if not diligently conducted. Additional objective measures of puberty, such as a physical examination, may help mitigate these issues. Given that the mean age for the APHV analysis was 12.3 years, which is relatively young for boys regarding attaining peak height velocity, we recommend that future analyses be conducted with boys at an older time point. Future studies could increase their power by collecting additional height samples over time per participant, oversampling extremes of pubertal onset timing (early and late), and with an in-depth examination of clinically significant pubertal timing differences.

While our sample is racially diverse, it included a more modest representation of children whose parents identify them as Hispanic. The socioeconomic status of our study population is likely more representative than many clinic-based studies of ADHD; however, the parental demographic of the sample is more likely to have both higher educational attainment and higher income compared with the national population. While the ECHO cohort can be considered more representative of a community sample compared with a clinical sample, some cohorts do oversample for medical diagnoses (e.g., asthma, obesity, neurodevelopmental disorders), which likely led to some higher prevalence rates than in the sample composition. For example, our sample had a higher rate of ADHD diagnosis via parent report than would be expected from the general population in the U.S. (American Psychiatric Association, 2013; Danielson et al., 2018; Willcutt, 2012). In a recent survey of parents of children 2–17 years of age in the U.S. (Danielson et al., 2018), 9.4% of parents reported that their child had been given an ADHD diagnosis, whereas 16.6% of the parents in our sample reported an ADHD diagnosis. The enriched sample, however, gave us greater power to explore the predictability of early ADHD symptoms on later outcomes (see Supplemental Materials for further discussion). An additional limitation of this study is the absence of self-ratings of internalizing symptoms, which may lead to underrepresentation of symptoms. In addition, we note that our analysis used symptom reports for both the early and outcome variables and therefore cannot address whether the children’s behavior would have met the criteria for a diagnosis, which requires a more comprehensive evaluation beyond parent rating. Further, the diagnostic information and subsequent data are snapshots in time, which may not be representative or indicative of symptomatology across time. These data considered a wide age range (early years: 3–9 years; outcome: 9–18 years), which may limit the interpretation of the outcomes. Future studies may also want to measure early manifestations of internalizing and externalizing symptoms in addition to early ADHD symptoms. Our analysis did not include those early variables due to harmonization-related issues of the measurements across multiple sites for the early time point. Relatedly, because this analysis involved a variety of participating studies, they may have differed in the specific assessment procedures and their timing. The heterogeneity of these procedures may have resulted in an underestimation of the true effects.

For future directions, a follow-up of this sample would be beneficial as the average age of our participants was about 12 years for the outcomes assessment. In addition, future studies may evaluate narrower age spans at both the early and outcome points. A different picture with the cohorts may emerge when they mature to an average age of 16 or 18 years. Ultimately, lifespan trajectory studies of this sample will be critical to understanding if elevated, early ADHD symptoms, as well as early anxiety, depression, oppositionality, and conduct symptoms, lead to later internalizing and externalizing symptoms throughout adulthood and even other psychopathology, such as bipolar disorder and psychosis.

### Summary

Using a nationwide sample of parent reports of ADHD symptoms in early development, this analysis revealed that early ADHD symptoms lead to an increase in later internalizing and externalizing symptoms based on parent report for both girls and boys. An early age of maximum growth in girls was associated with an increased likelihood of later Conduct Disorder, and there was a significant effect modification for APHV on ADHD that differed between boys and girls. To the extent these findings are confirmed, clinicians are encouraged to monitor the possibility that children with elevated symptoms of ADHD will need interventions to prevent and address a broad range of psychiatric symptoms as the child matures.

## Supplementary Material

1

## Figures and Tables

**Table 1.** Assessment and measured domains used to create early childhood and subsequent outcome scores

Analysis domain	CBCL^a^	BASC^b^	CPRS-3^c^	PROMIS^d^
ADHD symptoms	Attention-deficit/ hyperactivity problems*	Attention problems and/or hyperactivity	ADHD Index (10-item)	
Depression	Depressive problems*	Depression		Depressive Symptoms 6a – Parent Proxy
Anxiety	Anxiety problems*	Anxiety		Anxiety 8a – Parent Proxy
Aggressive behavior	Aggressive behavior	Aggression		
Conduct problems	Conduct problems*	Conduct problems		
Oppositional defiant problems	Oppositional defiant problems*			
Rule-breaking behavior	Rule-breaking behavior			

ADHD = attention-deficit/hyperactivity disorder.

a Child Behavior Checklist, Preschool and School-Age.

b Behavioral Assessment System for Children, Second and Third Edition.

c Conners 3 Rating Scales.

d Patient-Reported Outcomes Measurement Information System v2.0. Cell frequencies were de-identified to prevent their calculation for cells with small numbers of individuals.

**Table 2.** Sociodemographic description of analytic sample

	All (*n* = 2818)	With APHV (*n* = 2160)	Census
**Age at early childhood** (mean/SD)	5.3 (1.3)	5.4 (1.2)	
**Age at outcome** (mean/SD)	11.9 (2.3)	12.3 (2.5)	
**Sex**			
Male	1448 (51.4%)	1112 (51.5%)	
with ADHD *T* > 65	138 (9.5%)	122 (11%)	
Female	1370 (48.6%)	1048 (48.5%)	
with ADHD *T* > 65	117 (8.5%)	101 (9.6%)	
**Race**			
White	1472 (52.2%)	1137 (52.6%)	61.2%
Black	898 (31.9%)	672 (31.1%)	12.3%
Other Single Race	77 (0.03%)	55 (0.03%)	15.9%
Multiple Race	330 (11.7%)	267 (12.4%)	10.6%
Missing	41 (1.5%)	29 (1.3%)	
**Ethnicity**			
Hispanic	< 275	< 205	19.5%
Non-Hispanic	2539 (90.1%)	1955 (90.5%)	80.5%
Missing	< 10	< 5	
**Income**			
$50,000 or more	1712 (60.8%)	1375 (63.7%)	61.6%
< $50,000	847 (30.1%)	643 (29.8%)	38.4%
Missing	259 (9.2%)	142 (6.6%)	
**Highest education level**			
Less than high school	166 (5.9%)	135 (6.2%)	11.4%
High school degree, GED, or equivalent	433 (15.4%)	351 (16.2%)	26.9%
Some college or trade school	660 (23.4%)	538 (24.9%)	28.6%
Bachelor’s degree (BA, BS)	721 (25.6%)	523 (24.2%)	20.3%
Master’s degree or higher	738 (26.2%)	528 (24.4%)	12.8%
Missing	100 (3.5%)	85 (3.9%)	

APHV = age at peak height velocity; BA = Bachelor of Arts; BS = Bachelor of Science; GED = general education development.

**Table 3.** Associations between early-childhood ADHD symptoms and subsequent internalizing and externalizing symptoms (Model A)

		Anxiety				Depression		
Sex		Estimate (95% CI)		*N*		Estimate (95% CI)		*N*

Female		**0.19 (0.15,0.23)**		1359		**0.17 (0.13,0.21)**		1354

Male		**0.15 (0.11,0.19)**		1417		**0.16 (0.12,0.20)**		1416

	Aggressive behavior		Conduct problems		Oppositional defiant problems		Rule-breaking behavior	
Sex	Estimate (95% CI)	*N*	Estimate (95% CI)	*N*	Estimate (95% CI)	*N*	Estimate (95% CI)	*N*

Female	**0.23 (0.19,0.27)**	1363	**0.23 (0.19,0.27)**	1352	**0.18 (0.14,0.21)**	1288	**0.20 (0.17,0.23)**	1294

Male	**0.20 (0.16,0.24)**	1441	**0.16 (0.12,0.20)**	1409	**0.15 (0.12,0.19)**	1310	**0.14 (0.10,0.17)**	1339

*Note*. Exposures and outcomes are both expressed on normed *T*-score metrics; thus, coefficients can be interpreted on a Cohen’s *d* metric (i.e., expected SD increase in outcome per SD increase in independent variable) with respect to the respective norming samples of the measures.

**Table 4.** Associations between early-childhood attention-deficit/hyperactivity disorder (ADHD) symptoms, age at peak height velocity (APHV), their interaction, and subsequent internalizing and externalizing symptoms (Model B)

		Anxiety		Depression	
Sex	Coefficient	Estimate (95% CI)	*N*	Estimate (95% CI)	*N*

Female	ADHD	**0.16 (0.10,0.22)**	1037	**0.16 (0.11,0.21)**	1033

	APHV	0.12 (−0.69,0.93)	1037	−0.09 (−0.80,0.63)	1033

	APHV * ADHD	0.01 (−0.10,0.11)	1037	0.04 (−0.05,0.13)	1033

Male	ADHD	**0.12 (0.07,0.18)**	1082	**0.19 (0.13,0.24)**	1081

	APHV	−0.23 (−0.70,0.25)	1082	0.37 (−0.06,0.81)	1081

	APHV * ADHD	−0.02 (−0.07,0.03)	1082	0.06 (0.01,0.10)	1081

		Aggressive behavior		Conduct problems		Oppositional defiant problems		Rule-breaking behavior	
Sex	Coefficient	Estimate (95% CI)	*N*	Estimate (95% CI)	*N*	Estimate (95% CI)	*N*	Estimate (95% CI)	*N*

Female	ADHD	**0.16 (0.11, 0.21)**	1044	**0.18 (0.13, 0.23)**	1033	**0.12 (0.07, 0.16)**	973	**0.16 (0.12, 0.21)**	981

	APHV	−0.67 (−1.35, 0.02)	1044	**−0.76 (−1.45, −0.07)**	1033	−0.19 (−0.80,0.42)	973	−0.44 (−1.02, 0.15)	981

	APHV * ADHD	**−0.10 (−0.18, −0.01)**	1044	−0.07 (−0.15, 0.02)	1033	−0.07 (−0.15, 0.00)	973	−0.05 (−0.13, 0.02)	981

Male	ADHD	**0.23 (0.18, 0.28)**	1106	**0.17 (0.13, 0.22)**	1076	**0.17 (0.12, 0.22)**	988	**0.14 (0.09, 0.18)**	1016

	APHV	0.04 (−0.37, 0.44)	1106	0.20 (−0.19, 0.60)	1076	0.08 (−0.33,0.48)	988	0.14 (−0.21, 0.49)	1016

	APHV * ADHD	**0.06 (0.02, 0.11)**	1106	**0.05 (0.01, 0.09)**	1076	0.05 (0.00,0.09)	988	0.03 (−0.01, 0.06)	1016

*Note.* The results for associations with confidence intervals that exclude zero are presented in bold. Exposures and outcomes are both expressed on normed *T-*score metrics; thus, coefficients can be interpreted on a Cohen’s *d* metric with respect to the respective norming samples of the measures.

## Data Availability

De-identified data from the ECHO Program are available through NICHD’s Data and Specimen Hub (DASH) (https://dash.nichd.nih.gov). DASH is a centralized resource that allows researchers to access data from various studies via a controlled-access mechanism. Researchers can now request access to these data by creating a DASH account and submitting a Data Request Form. The NICHD DASH Data Access Committee will review the request and provide a response in approximately two to three weeks. Once granted access, researchers will be able to use the data for three years. See the DASH Tutorial for more detailed information on the process (https://dash.nichd.nih.gov/resource/tutorial).
