# Supplementary material for: Prediction of internalizing and externalizing symptoms in late childhood from attention-deficit/hyperactivity disorder symptoms in early childhood
Source: Dev Psychopathol. Author manuscript; Available in PMC 2025 Nov 1. (PMC11427612; doi:10.1017/S0954579424000695)
Supplement: 1 [file NIHMS1972681-supplement-1.docx]

**Supplemental material**

**Time between Initial ADHD Symptomatology Assessment and Outcome Assessment**

Table S1 contains means and standard deviations of the time between initial ADHD symptomatology assessment and subsequent outcome assessment, separated by outcome domain and analysis sample.

| **Table S1: Means and standard deviations of the time between initial ADHD symptomatology assessment and subsequent outcome assessment for full and APHV subsample.** | | | | | | | | | |
| --- | --- | --- | --- | --- | --- | --- | --- | --- | --- |
|  | |  | |  | |  | |  | |
|  | | Full Sample | | | | APHV Subsample | | | |
| Outcome Domain | Mean Years btw Baseline & Follow-Up | | SD Years btw Baseline & Follow-Up | | Mean Years btw Baseline & Follow-Up | | SD Years btw Baseline & Follow-Up | |  |
| Aggressive Behavior | 6.59 | | 2.75 | | 6.88 | | 2.90 | |  |
| Anxiety | 6.60 | | 2.76 | | 6.90 | | 2.91 | |  |
| Anxious/Depressed | 6.71 | | 2.79 | | 7.05 | | 2.93 | |  |
| Conduct Problems | 6.61 | | 2.76 | | 6.91 | | 2.91 | |  |
| Depression | 6.60 | | 2.76 | | 6.91 | | 2.91 | |  |
| Oppositional Defiant | 6.74 | | 2.79 | | 7.09 | | 2.94 | |  |
| Rule Breaking Behavior | 6.71 | | 2.79 | | 7.05 | | 2.93 | |  |
| Withdrawn/Depressed | 6.71 | | 2.79 | | 7.05 | | 2.93 | |  |
|  | |  | |  | |  | |  | |

**Representativeness of Study Sample for Demographic Variables**

Compared with the 2021 United States (U.S.) Census data (see Table 1 in main text), our sample had a higher percentage of Black individuals (31.1-31.9% for the two analysis samples) and a lower percentage of individuals of any single race other than White and Black (<50 individuals) than the general U.S. population (12.3% and 15.9%, respectively) (US Census Bureau, 2021a). Our sample similarly underrepresented Hispanic individuals, with 90.1-90.5% of our sample identifying as non-Hispanic compared with 80.5% of the general U.S. population (US Census Bureau, 2021b). Income and maternal education levels in our sample were roughly comparable to the general U.S. population (US Census Bureau, 2021c; US Census Bureau, 2021d). Few data were missing for these variables; therefore, these numbers can be compared directly.

**Representativeness of Study Sample for the Prevalence of Neurodevelopmental Diagnoses**

Table S2 shows neurodevelopmental diagnoses reported at any time during a participant’s lifetime to add to the description of this population. Between 20% and 40% of children had missing information for each of these diagnoses: 9.9% of caregivers reported a diagnosis of anxiety; 4.3% reported a diagnosis of depression; and 3% reported a diagnosis of autism, autism spectrum disorder, or pervasive developmental disorder; note that an individual could receive multiple diagnoses. The proportion reporting a diagnosis of ADHD was relatively high, at 13.7%. Developmental delay was reported by 4.9%, intellectual disability by 1.3%, and learning disability by 7.2%. The proportions of participants reporting each of these diagnoses were similar among the study sample included in the age at peak height velocity (APHV) analysis.

**Table S2: Neurodevelopmental diagnoses among study participants**

|  | **All  (n=2818)** | **With APHV  (n=2160)** |
| --- | --- | --- |
| **Anxiety** |  |  |
| Yes | 280(9.9%) | 241(11.2%) |
| No | 1850(65.6%) | 1401(64.9%) |
| Missing | 688(24.4%) | 518(24%) |
| **Depression** |  |  |
| Yes | 122(4.3%) | 111(5.1%) |
| No | 1994(70.8%) | 1517(70.2%) |
| Missing | 702(24.9%) | 532(24.6%) |
| **Autism, ASD, or PDD** |  |  |
| Yes | 85(3%) | 61(2.8%) |
| No | 2089(74.1%) | 1595(73.8%) |
| Missing | 644(22.9%) | 504(23.3%) |
| **“ADD” or ADHD** |  |  |
| Yes | 386(13.7%) | 324(15%) |
| No | 1785(63.3%) | 1332(61.7%) |
| Missing | 647(23%) | 504(23.3%) |
| **Developmental delay** |  |  |
| Yes | 138(4.9%) | 104(4.8%) |
| No | 1670(59.3%) | 1410(65.3%) |
| Missing | 1010(35.8%) | 646(29.9%) |
| **Intellectual disability** |  |  |
| Yes | 36(1.3%) | 29(1.3%) |
| No | 1788(63.4%) | 1481(68.6%) |
| Missing | 994(35.3%) | 650(30.1%) |
| **Learning disability** |  |  |
| Yes | 203(7.2%) | 163(7.5%) |
| No | 1913(67.9%) | 1468(68%) |
| Missing | 702(24.9%) | 529(24.5%) |

ADD, attention-deficit disorder; ADHD, attention-deficit/hyperactivity disorder; APHV, age at peak height velocity; ASD, autism spectrum disorder; PPD, pervasive developmental disorder.

With respect to mental conditions (Table S2), we can compare the prevalence in our study samples to population estimates by looking only at the proportions of the complete data that were diagnosed with each condition. Compared with estimates from Zablotsky et al. (2017), our sample had higher rates of autism, autism spectrum disorder (ASD), and pervasive developmental disorder (PDD) (3.7-3.9% compared with 2.8%); developmental delay (6.9-7.6% compared with 4.6%); and intellectual disability (1.9-2% compared with 1.1%) than those in the general U.S. population. The National Center for Health Statistics Data Query Tool lists the estimated rate of learning disability in the U.S. child population as 6.6% (National Center for Health Statistics, 2019)^.^ Compared with this reference, our sample had a higher rate of learning disability (9.6-10.0%) than the general U.S. population. Other sources found through the Centers for Disease Control and Prevention (CDC) website report the prevalence of the remaining mental disorders in Table S2. Compared with these numbers, our sample had higher rates of anxiety and depression (13.1-14.7% compared with 7.1% and 5.8-6.8% compared with 3.2%, respectively) (Bitsko et al., 2018) and attention-deficit disorder (ADD) and attention-deficit/hyperactivity disorder (ADHD) (17.8-19.6% compared with 9.4%) (Danielson et al., 2016) than the U.S. child population.

We note that because the diagnoses in our analysis were based on parent report rather than reports from physicians or mental health clinicians, we could not confirm a formal diagnosis of ADHD or of any of the other disorders. In addition, we could not determine whether the gold standard methods for diagnosing ADHD were used and whether the participants met the diagnostic criteria for ADHD. The ECHO Program is collecting those data and hopefully future reports will be able to verify clinical diagnoses.

Table S3a shows the frequencies of all combinations of measures used in the full sample and in Table S3b for the APHV subsample.

**Table S3a: Forms used to assess exposures and outcomes: full sample (N=2818)**

| **Domain** | **Set 1** | | **Set 2** | **Set 3** | **Set 4** | **Set 5** | **Set 6** | **Set 7** | **Set 8** |
| --- | --- | --- | --- | --- | --- | --- | --- | --- | --- |
| Aggressive Behavior | CBCL | | CBCL | CBCL | BASC | CBCL | CBCL |  | CBCL |
| Anxiety | CBCL | | CBCL | CBCL | BASC |  | CBCL | PROMIS | CBCL |
| Anxious Depressed | CBCL | | CBCL | CBCL |  | CBCL | CBCL |  | CBCL |
| Conduct Problems | CBCL | | CBCL | CBCL | BASC |  | CBCL |  | CBCL |
| Depression | CBCL | | CBCL | CBCL | BASC |  | CBCL | PROMIS | CBCL |
| Oppositional Defiant | CBCL | | CBCL | CBCL |  |  | CBCL |  | CBCL |
| Rule-Breaking | CBCL | | CBCL | CBCL |  | CBCL | CBCL |  |  |
| Withdrawn Depressed | CBCL | | CBCL | CBCL |  | CBCL | CBCL |  | CBCL |
| ADHD | CBCL | | CADHD | CBCL | BASC | CBCL | BASC | CBCL | CBCL |
| Attention | CBCL | |  | CBCL | BASC | CBCL | BASC | CBCL | CBCL |
| Hyperactivity Impulsivity | |  |  | BASC | BASC |  | BASC |  |  |
| Number of Participants | 1763 | | 411 | 380 | 167 | 41 | 16 | 8 | 5 |

ADHD, attention-deficit/hyperactivity disorder; APHV, age at peak height velocity; BASC, Behavior Assessment System for Children; CBCL, Child Behavior Checklist.

**Table S3b: Forms used to assess exposures and outcomes: APHV subsample (N=2160)**

| **Domain** | **Set 1** | | **Set 2** | **Set 3** | **Set 4** | **Set 5** | **Set 6** | **Set 7** |
| --- | --- | --- | --- | --- | --- | --- | --- | --- |
| Aggressive Behavior | CBCL | | CBCL | CBCL | BASC | CBCL | CBCL |  |
| Anxiety | CBCL | | CBCL | CBCL | BASC |  | CBCL | PROMIS |
| Anxious Depressed | CBCL | | CBCL | CBCL |  | CBCL | CBCL |  |
| Conduct Problems | CBCL | | CBCL | CBCL | BASC |  | CBCL |  |
| Depression | CBCL | | CBCL | CBCL | BASC |  | CBCL | PROMIS |
| Oppositional Defiant | CBCL | | CBCL | CBCL |  |  | CBCL |  |
| Rule-Breaking | CBCL | | CBCL | CBCL |  | CBCL | CBCL |  |
| Withdrawn Depressed | CBCL | | CBCL | CBCL |  | CBCL | CBCL |  |
| ADHD | CBCL | | CADHD | CBCL | BASC | CBCL | BASC | CBCL |
| Attention | CBCL | |  | CBCL | BASC | CBCL | BASC | CBCL |
| Hyperactivity Impulsivity | |  |  | BASC | BASC |  | BASC |  |
| Number of Participants | 1204 | | 409 | 321 | 152 | 40 | 7 | 5 |

ADHD, attention-deficit/hyperactivity disorder; BASC, Behavior Assessment System for Children; CBCL, Child Behavior Checklist.

**Proportion of Participants with Outcome Assessments in Regard to their Estimated APHV**

Table 4 shows proportions of males and females with outcome assessments after their estimated APHV for each outcome and analysis sample.

| **Table S4. Proportions of males and females with outcome assessments after their estimated APHV for each outcome and analysis sample,** | | | | | | | |  |
| --- | --- | --- | --- | --- | --- | --- | --- | --- |
|  |  |  |  |  |  | |  |  |
|  |  | Full Sample | | | | APHV Subsample | | |
| Child Sex | Outcome Domain | *N with APHV<=Outcome | **N with Outcome>APHV | ***Proportion with Outcome After APHV | ^N with APHV<=Outcome | | ^^N with Outcome>APHV | ^^^Proportion with Outcome After APHV |
| Female | Aggressive Behavior | 216 | 827 | 0.793 | 216 | | 827 | 0.793 |
|  | Anxiety | 218 | 818 | 0.790 | 218 | | 818 | 0.790 |
|  | Anxious/Depressed | 216 | 766 | 0.780 | 216 | | 766 | 0.780 |
|  | Conduct Problems | 216 | 816 | 0.791 | 216 | | 816 | 0.791 |
|  | Depression | 216 | 816 | 0.791 | 216 | | 816 | 0.791 |
|  | Oppositional Defiant | 215 | 757 | 0.779 | 215 | | 757 | 0.779 |
|  | Rule Breaking Behavior | 216 | 764 | 0.780 | 216 | | 764 | 0.780 |
|  | Withdrawn/Depressed | 215 | 765 | 0.781 | 215 | | 765 | 0.781 |
| Male | Aggressive Behavior | 681 | 426 | 0.385 | 680 | | 426 | 0.385 |
|  | Anxiety | 663 | 420 | 0.388 | 662 | | 420 | 0.388 |
|  | Anxious/Depressed | 613 | 404 | 0.397 | 612 | | 404 | 0.398 |
|  | Conduct Problems | 658 | 419 | 0.389 | 657 | | 419 | 0.389 |
|  | Depression | 662 | 420 | 0.388 | 661 | | 420 | 0.389 |
|  | Oppositional Defiant | 591 | 398 | 0.402 | 590 | | 398 | 0.403 |
|  | Rule Breaking Behavior | 613 | 404 | 0.397 | 612 | | 404 | 0.398 |
|  | Withdrawn/Depressed | 613 | 404 | 0.397 | 612 | | 404 | 0.398 |
| *Column presents numbers of children whose age at peak height velocity (APHV) was before or at the same time as the age at outcome data collection ("APHV <= Outcome") from the full sample; **Column presents number whose APHV was after the age at outcome data collection ("Outcome<APHV") for the full sample; ***Column presents the proportion of children who were past the APHV at the time of follow-up for the full sample. ^Column presents numbers of children whose APHV was before or at the same time as the age at outcome data collection for the APHV subsample; ^^Column presents the proportion of children who were past the APHV at the time of follow-up for the APHV subsample; ^^^Column presents the proportion of children who were past the APHV at the time of follow-up for the APHV subsample. | | | | | | | | |
|  |  |  |  |  |  | |  |  |
|  |  |  |  |  |  | |  |  |

**REFERENCES**

Bitsko, R. H., Holbrook, J. R., Ghandour, R. M., Blumberg, S. J., Visser, S. N., Perou, R., Walkup, J. (2018). Epidemiology and impact of healthcare provider diagnosed anxiety and depression among US children. *Journal of Developmental and Behavioral Pediatrics*, 39(5), 395-403.

Danielson, M. L., Bitsko, R. H., Ghandour, R. M., Holbrook, J. R., Blumberg, S. J. (2018) Prevalence of parent-reported ADHD diagnosis and associated treatment among U.S. children and adolescents, 2016. Journal of Clinical Child and Adolescent Psychology, 47(2), 199-212.

National Center for Health Statistics, 2019: National Center for Health Statistics. Percentage of ever having a learning disability for children aged 3-17 years, United States, 2019. National Health Interview Survey. Generated interactively: Dec 06 2021 from https://wwwn.cdc.gov/NHISDataQueryTool/SHS_2019_CHILD3/index.html

US Census Bureau, 2021a: https://data.census.gov/cedsci/table?tid=DECENNIALPL2020.P1

US Census Bureau, 2021b: https://data.census.gov/cedsci/table?tid=DECENNIALPL2020.P2

US Census Bureau, 2021c: https://data.census.gov/cedsci/table?tid=ACSST1Y2019.S1901

US Census Bureau, 2021d: https://data.census.gov/cedsci/table?tid=ACSST1Y2019.S1501

Zablotsky, B., Black, L.I., & Blumberg, S.J. (2017). Estimated prevalence of children with diagnosed developmental disabilities in the United States, 2014-2016. NCHS Data Brief, No. 291. Retrieved from https://www.cdc.gov/nchs/data/databriefs/db291.pdf
